# Combination of a GnRH agonist with an antagonist prevents flare-up effects and protects primordial ovarian follicles in the rat ovary from cisplatin-induced toxicity: a controlled experimental animal study

**DOI:** 10.1186/1477-7827-11-16

**Published:** 2013-03-01

**Authors:** Xiaoyan Li, Xiang Kang, Qingchun Deng, Jing Cai, Zehua Wang

**Affiliations:** 1 Department of Obstetrics and Gynecology, Union hospita, Tongji Medical College, Huazhong University of Science and Technology, Wuhan, China

**Keywords:** GnRH agonist, GnRH antagonist, Chemotherapy, Ovarian protection

## Abstract

**Background:**

With the continuous improvement of surgery and chemotherapeutic treatments, many tumour patients increasingly achieve long-term survival and can even be completely cured. However, platinum-containing drugs, which are widely used to treat a variety of types of cancer, cause menstrual disorders and ovarian failure, which in turn lead to infertility. Thus far, gonadotropin releasing hormone (GnRH) agonist (GnRHa) and antagonist (GnRHant) are reported to act as protective agents of the ovary in chemotherapy through the inhibition of the female gonadal axis. Nevertheless, they both have disadvantages that limit their use. GnRHa causes a flare-up effect during the first week after administration, and no long-acting GnRHant agent is available. GnRHa combined with GnRHant may prevent the flare-up effect of GnRHa and rapidly inhibit the female gonadal axis. Several clinical studies with small sample sizes have reported controversial conclusions. In this strictly controlled animal study, we investigated the advantages of combination treatment with GnRHa and GnRHant.

**Methods:**

Rats aged 12 weeks were divided into six groups: Control, cisplatin (CDDP), GnRHa, GnRHant, Combination (sht, short-term) and Combination (lng, long-term) of GnRHa and GnRHant. The last four groups received Triptorelin (1 mg/kg·d, for 14 days), Cetrorelix (0.5 mg/kg·d, for 10 days), a combination of Triptorelin (1 mg/kg·d, for 10 days) and Cetrorelix (0.5 mg/kg·d, for 10 days) in the long-term group and for 3 days in the short-term group. The Control and CDDP groups received saline (1 ml/kg·d, for 10 day). Then, all groups apart from the Control group received cisplatin (1 mg/kg·d, for 10 days), and the Control group received another 10 days of saline as described above. Blood samples were collected to detect the serum levels of E2, LH and FSH. Observation of oestrous cyclicity was also performed after drug administration. Finally, bilateral ovaries were collected for histological study and follicle counting.

**Results:**

We observed a flare-up effect in rats treated with GnRHa, but not in any of the combination groups. The percentage of normal cyclicity increased from 0% in the CDDP group to 25.0%, 33.3%, 66.7% and 41.7%, in the GnRHa, GnRHant, combination (lng) and combination (sht) groups, respectively. Pretreatment with GnRHa, GnRHant and combination (lng) significantly protected the primordial follicles from destruction by preserving 57.6%, 63.4%, 87.1% and 60.4% of the follicles, respectively.

**Conclusions:**

The combination of a GnRH agonist with antagonist completely prevented the flare-up effect and enhanced the protective effect of the ovary from cisplatin-induced gonadotoxicity in rats.

## Background

With the continuous improvement of surgical and chemotherapeutic treatments, many tumour patients achieve long-term survival and can even be completely cured [1]. Platinum-containing anti-cancer drugs are widely used to treat a variety of cancers, including sarcomas, carcinomas, and lymphomas. Cis-diamminedichloroplatinum (cisplatin, CDPP) is the first and most representative drug in this class. Cisplatin-based combination chemotherapy displays significant antitumor activity against cancers of the testis, ovary, head, neck and lung. The underlying mechanism is mainly due to chemical bonding to DNA, leading to crosslinking of the DNA, which induces cell apoptosis [2,3]. However, cisplatin may also harm the granulosa cells of the follicles, causing menstrual disorders and acute or chronic ovarian failure, resulting in infertility [4,5]. Gynaecological oncologists and researchers are faced with the challenge of protecting ovaries from damage caused by chemotherapy and improving the quality of life for patients [6,7].

Natural gonadotropin-releasing hormone (GnRH) is a short-acting decapeptide that is secreted by the hypothalamus, which could induce the secretion of LH and FSH in the pituitary gland [8]. A modification in the 6th and 10th amino acids of GnRH results in the GnRH agonist (GnRHa), which has increased biological activity compared to natural GnRH. The inhibition of the female gonadal axis by GnRHa could reduce the damage caused to primordial follicles by chemotherapeutic agents [9]. Nevertheless, the initial flare-up effect that occurs during the first week of GnRHa treatment limits its use [10].

GnRH antagonist (GnRHant) is one of the derivatives of GnRHa, and it has a significant protective effect on ovarian function through a stronger and rapid inhibition of the female gonadal axis. However, the effect is short lived. No long-acting antagonist is available [9,11]. Several clinical studies with small sample sizes have suggested that GnRHant combined with GnRHa could prevent the flare-up effect of GnRHa and rapidly inhibit the female gonadal axis [12-15]. However, it is difficult to obtain a strict negative control in clinical studies. In the present study, using animal models, we investigated the advantages of combined treatment with GnRHa and GnRHant for weakening the GnRHa-induced flare-up effect and protecting the ovary from the damage induced by CDPP.

## Methods

### Animals

Sexually mature female Wistar rats (12 weeks old, body weight 280±20 g) were obtained from the Disease Prevention and Control Center of Hubei Province, China, and housed under special pathogen-free conditions. The animals were kept in individual plastic cages with ad libitum access to full-price feed and water in a temperature-controlled room (22±2°C) on a 12 h light, 12 h dark schedule. All of the experimental procedures were performed at the experimental animal centre of Tongji Medical College, Huazhong University of Science and Technology (HUST), China, according to the international ethical guidelines and with the approval of the HUST Ethics Committee.

### Drug treatment

A total of 72 rats with normal oestrous cycles (4–5 days) were randomly divided into six groups: Control, CDDP, GnRHa, GnRHant combination (lng, long-term) and combination (sht, short-term). The control group received 1 ml/kg·d of saline for 20 days. The CDDP group received 1 ml/kg·d of saline for 10 days followed by 1 mg/kg·d of cisplatin (Qilu Pharmaceuticals Co. LTD, China) for 10 days. The GnRHa group received depot injections of 1 mg/kg of Triptorelin (Diphereline, Beaufour-Ipsen Pharmaceutical Co. LTD., Tianjin) followed two weeks later by 1 mg/kg·d cisplatin for 10 days. The GnRHant group received 0.5 mg/kg·d of Cetrorelix subcutaneously (Owto biotech INC. China) for 10 days followed by 1 mg/kg·d of cisplatin for 10 days. The combination (lng) group received depot injection of 1 mg/kg of Triptorelin in combination with 0.5 mg/kg·d of Cetrorelix for 10 days followed by cisplatin as mentioned above. The combination (sht) group was only co-treated with Cetrorelix for 3 days. All of the other agents were administered intraperitoneally. Pretreatment with the GnRH analogues was performed at 9 am every day, and cisplatin was injected at 11 am because previous studies have shown that the serum concentration of Cetrorelix reached a peak 1–2 h after injection.

### Estradiol (E2), Luteinising Hormone (LH) and Follicle-Stimulating Hormone (FSH) assays

Blood specimens were collected on the 1st, 3rd, 5th and 8th day of the treatment schedule through the orbital vein. Serum levels of E2, LH and FSH were analysed by radioactive assay kits (Beijing North Institute of Biological Technology, China) following the manufacturer’s instructions.

### Observation of oestrous cyclicity

The oestrous cycle was monitored for 10 days after the treatment. Smears were obtained by carefully inserting a non-needle syringe into the vagina and flushing with approximately 0.2 ml saline. The washes were spotted on slides, and then the air-dried smears were stained with haematoxylin. The slides were examined under a microscope to determine the phase of the oestrous cycle. The different stages of the oestrous cycle were determined according to the predominant cell type present in the vaginal smears by light microscopy [16]. Normal oestrous cyclicity was defined as the occurrence of at least two consecutive normal oestrous cycles lasting for 4–5 days with 1–2 days of oestrus. The cycle length was defined as the number of consecutive days between the oestrus smears in the previous and the subsequent cycles.

### Histological study and follicle counting

Bilateral rat ovaries were taken after observation of the oestrous cyclicity and fixed in 4% paraformaldehyde overnight. Then, the ovaries were embedded in paraffin and cut into 5-μm serial sections to be stained by haematoxylin and eosin (HE). Every tenth section (50 slices in average per ovary) was selected for observation under a light microscope (IX71, Olympus Optical Co. LTD, Japan). The scoring system offered by Sağsöz et al. [17], was used for histopathological evaluation of the ovarian tissues, with some modifications. The histological sections were examined for the presence of haemorrhage, cortical fibrosis, follicular atresia and blood vessel damage. The changes were scored from 0 to 3 according to their severity, where 0 represents no pathological finding, and 1, 2, and 3 represent pathological findings of < 33%, 33–66%, and > 66% of the ovary, respectively. The scores for each parameter were summed and the total scores were calculated. The follicles were classified into three stages as follows: primordial, growing (primary and secondary) and mature follicles. A primordial follicle contains a partial or complete layer of flattened granulosa cells encircling the oocyte. In the primary follicle, the oocyte is surrounded by a single layer of cuboidal granulosa cells. The secondary follicle contains multiple layers of cuboidal granulosa cells surrounding the oocyte with little or no antral space. A mature follicle contains a single large antral space adjacent to the oocyte. The summation of follicles in different stages was calculated for analysis.

### Statistical analysis

SPSS 11.0 (SPSS Inc., Chicago, Illinois, USA) was used for statistical analysis. One-Way ANOVA was performed to analyse the quantitative data followed by the LSD-t test for multiple comparison. Categorical data were analysed by the Kruskal-Wallis test, and multiple comparisons were performed using the Mann–Whitney U test. All tests of significance were two-sided, and the significance value was set at <0.05, whereas α’ was adjusted to 0.0017 in the Mann–Whitney U test.

## Results

### Combination pretreatment completely prevented gonadotropin flare-up

**Figure 1:**
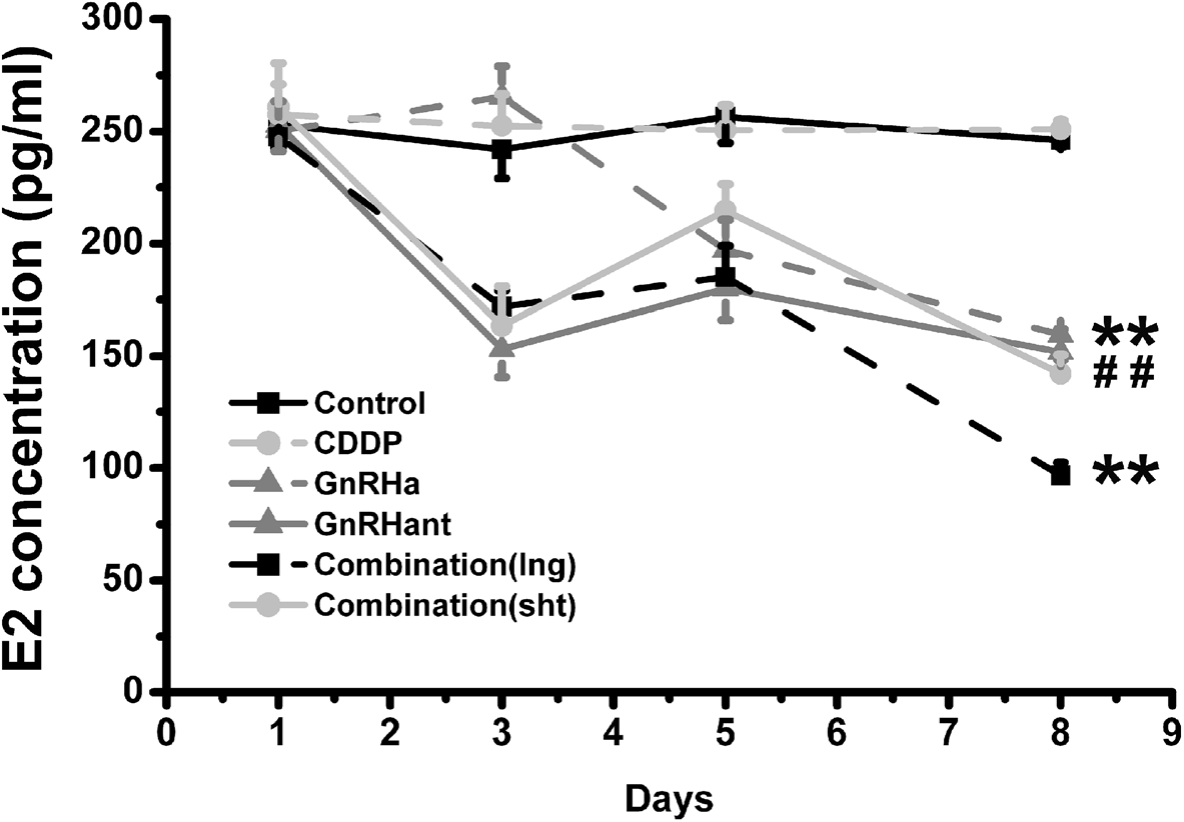
**Serum gonadotropin levels.** The average E2 levels during pretreatment in the control, GnRHa, GnRHant combination (lng) and combination (sht) groups. Blood samples were taken on the 1st, 3rd, 5th and 8th day. **P<0.01, compared with the control group; ##P<0.01, compared with the combination (lng) group.

Serum levels of E2 during pretreatment in the six groups are shown in Figure 1. GnRHant and both combination treatments significantly decreased the E2 level on day 3 compared to the control group (P<0.001), and GnRHa treatment slightly increased the E2 level. On day 5, the E2 level in the GnRHa group decreased. On day 8, all four treated groups had lower levels of E2, compared with the control group (P<0.001). Moreover, the combination (lng) group presented a significantly decreased level of E2 compared to that in either the GnRHa or GnRHant groups (P<0.001). No significant change was observed in FSH level. LH level significantly increased in the GnRHa group on day 3 compared with the other five groups (P<0.001). A detailed description of the FSH, LH and E2 levels in different groups at different time points is given in Table 1.

**Table 1:** Hormonal levels during the course of GnRH analogue pretreatment

**Groups**	**n**	**FSH (mIU/ml)**				**LH (mIU/ml)**				**E2 (pg/ml)**			
		**1st day**	**3rd day**	**5th day**	**8th day**	**1st day**	**3rd day**	**5th day**	**8th day**	**1st day**	**3rd day**	**5th day**	**8th day**
***Control***	12	2.45±0.13	2.43±0.08	2.32±0.06	2.45±0.12	2.77±0.18	2.83±0.19	2.84±0.11	2.90±0.21	252.98±10.29	242.00±12.96	256.60±11.75	246.03±2.80
***CDDP***	12	2.51±0.16	2.49±0.09	2.41±0.05	2.44±0.14	2.75±0.16	2.83±0.20	2.66±0.32	2.89±0.17	257.71±13.28	252.60±13.90	250.59±11.07	250.98±4.09
***GnRHa***	12	2.47±0.08	2.71±0.10	2.21±0.07	2.33±0.11	2.87±0.18	3.93±0.13^a^	2.81±0.27	2.89±0.11	250.34±13.01	265.42±13.49	197.10±13.43	159.63±2.52^ab^
***GnRHant***	12	2.33±0.07	2.22±0.11	2.01±0.07	2.14±0.14	2.70±0.12	2.56±0.06	2.42±0.14	2.62±0.08	252.83±11.75	153.04±12.37a	179.87±13.83	151.87±3.43^ab^
***Combination (lng)***	12	2.52±0.13	2.29±0.10	2.44±0.05	2.48±0.07	2.96±0.20	2.74±0.05	2.73±0.13	2.64±0.30	247.65±2.80	171.95±7.20a	185.10±13.86	96.57±5.79^a^
***Combination (sht)***	12	2.64±0.09	2.33±0.10	2.24±0.25	2.30±0.16	2.88±0.12	2.53±0.17	2.86±0.21	2.75±0.07	260.86±19.57	163.60±17.4a	214.63±11.77	142.21±8.34^ab^

^a^*P*<0.01 *vs.* control group; ^b^*P*<0.01 *vs.* combination (lng) group.

Data are expressed as the mean ± standard error.

### Combination pretreatment partly restored oestrous cyclicity

**Figure 2:**
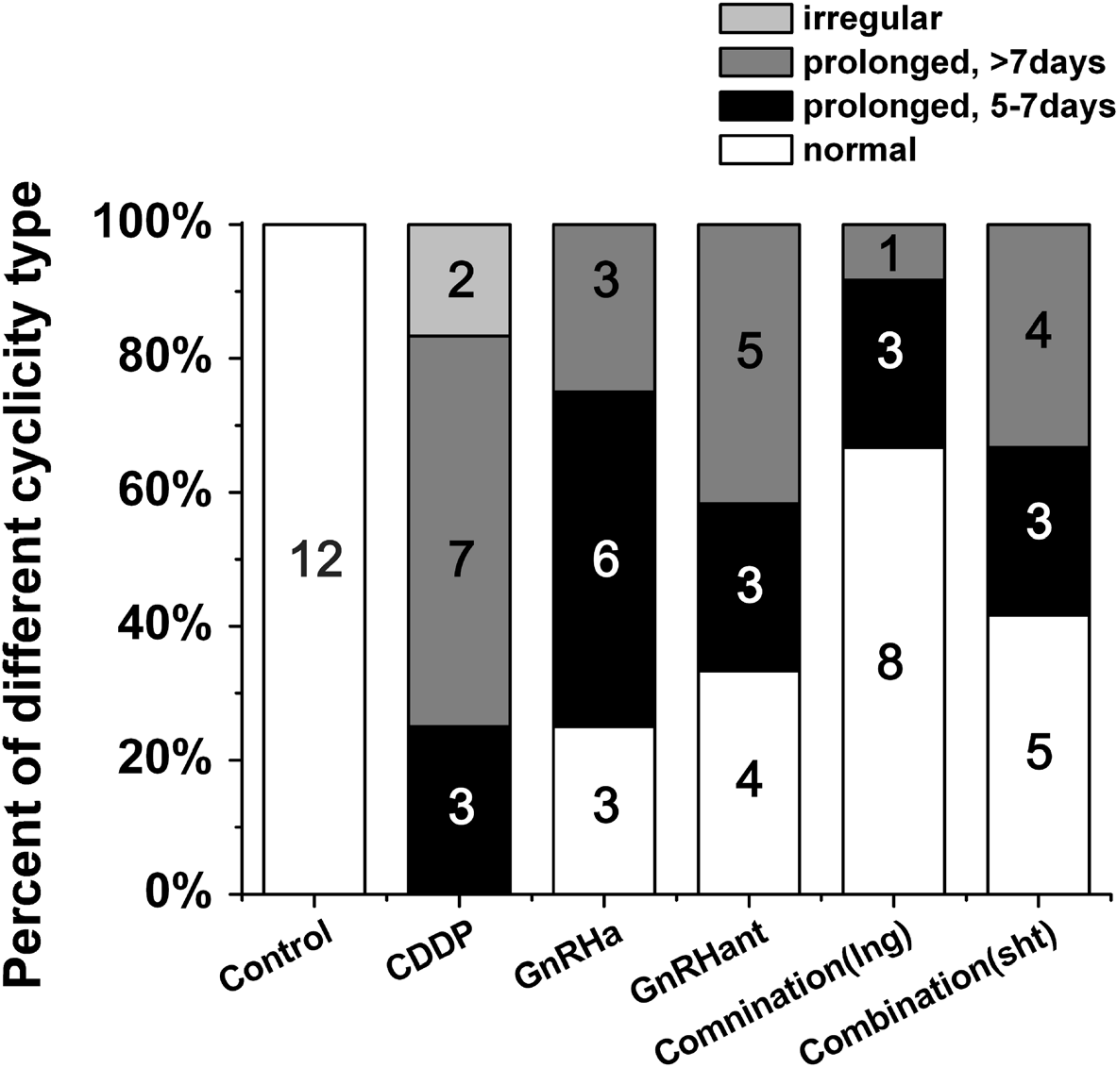
**Oestrous cyclicity.** The percentage of animals experiencing normal, prolonged or irregular cycles in the control, CDDP, GnRHa, GnRHant combination (lng) and combination (sht) groups. The numbers in the column indicate the numbers of rats.

Figure 2 shows the percentage of animals with normal, prolonged or irregular cycles. There were significant differences in the distribution of the different types of cyclicity between the groups (P<0.001). Rats in the control group all showed normal (4–5 days) oestrous cycles, while treatment with CDDP significantly induced either irregular oestrus or a prolonged cycle length that was more than 7 days in the majority of the animals (58.3%) (P<0.001). This cyclic change could be partly reversed by pretreatment with GnRHa, GnRHant or a combination of both. The percentage of normal cyclicity increased from 0% to 25.0%, 33.3%, 66.7% and 41.7%, respectively. Compared to the CDDP group, the combination (lng) group showed a significantly altered distribution of cyclicity (P<0.001). No such significance was found in the GnRHa, GnRHant or the combination (sht) group, but there were more (50%) rats experiencing a slightly prolonged cycle (5–7 days) in the GnRHa group (P_GnRHa_=0.007, P_GnRHant_=0.029, P_sht_=0.010).

### Combination pretreatment preserved more primordial follicles

**Figure 3:**
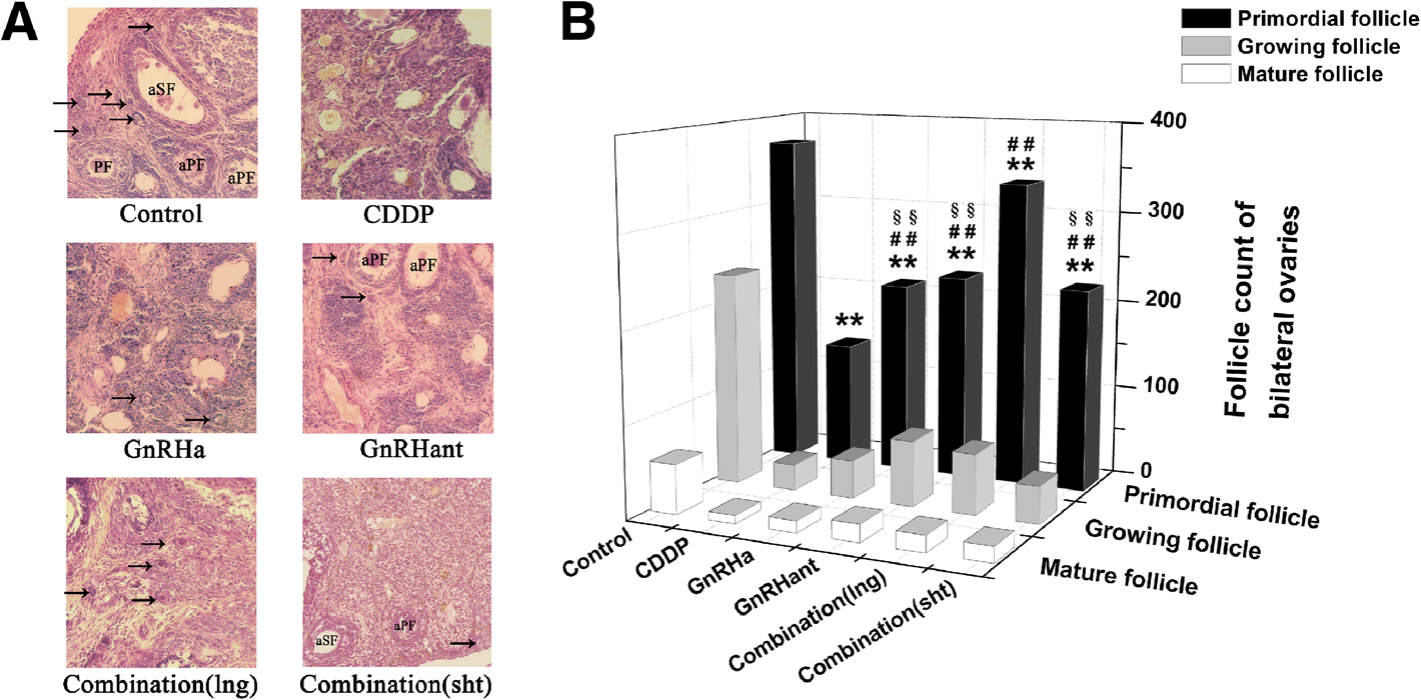
**Ovarian histological study and follicle counting.** Histological examination of ovaries, 100×. Numerous follicles with normal morphology in the ovaries of rats in the control group; an obvious decline of follicles and the destruction of the ovarian structure in the CDDP group; a remarkable sparing of primordial follicles in the GnRHa group, the GnRHant group and both combination groups. Arrow—primordial follicle; PF—primary follicle; aPF—atretic primary follicle; SF—secondary follicle; aSF—atretic primary follicle (**A**). Primordial, growing, and mature follicle counts of rats in the control, CDDP, GnRHa, GnRHant and combination groups. **P<0.01, compared to the control group; ## *P*<0.01, compared to the CDDP group; §§ *P*<0.01, compared to the combination (lng) group (**B**).

The follicles at different stages were observed under a light microscope. In the control group, the number of follicles at various stages was present and the layers of surrounding granulosa cells were integral. In the CDDP group, the follicle structure was destroyed, resulting in a remarkable reduction in number. Obvious damage including cortical fibrosis, follicular atresia and blood vessel damage induced by cisplatin was significantly different from that in control group (P<0.001, See Additional file 1: Figure S1). None of the pretreatments could rescue the tissue damage. More primordial follicles were reserved in other groups despite the pathological changes mentioned above (Figure 3A).

The follicles in each stage were counted, as shown in Figure 3B, and the detailed numbers are listed in Table 2. The differences between groups in primordial, growth and mature follicles were significant (P<0.001). CDDP induced a significant reduction of primordial, growing, mature and total follicles compared with the control group (P<0.001), and 63.2% of the primordial follicle pool was lost. The GnRHa, GnRHant, combination (lng) and combination (sht) groups showed significant protection of the primordial follicles from destruction, with preservation of 57.6%, 63.4%, 87.1% and 60.4%, respectively (P<0.001). In addition, the differences between the combination (lng) group and the single pretreatment groups or the combination (sht) group were also significant (P<0.001). The reduction of the growing and mature follicles was not reversed except in the GnRHant group, and the number of growing follicles in the GnRHant group differed significantly from that in the CDDP group.

**Table 2:** Follicle count of bilateral ovaries in the 6 groups

**Groups**	**n**	**Primordial follicle**	**Growing follicle**	**Mature follicle**	**Total**
***Control***	12	370.5±6.9	235.3±31.1	54.6±17.1	660±24.4
***CDDP***	12	136.5±23.1^a^	28.6±4.7^a^	9.1±6.3^a^	174.2±32.5^a^
***GnRHa***	12	213.2±22.3^abc^	41.6±12.3^a^	13±10.6^a^	267.8±34.2^abc^
***GnRHant***	12	227.5±8.9^abc^	71.5±12.0^ab^	19.5±6.8^ab^	318.5±6.9^abc^

^a^*P*<0.01 *vs.* control group; ^b^*P*<0.01 *vs.* CDDP group; ^c^*P*<0.01 *vs.* combination (lng) group; ^d^*P*<0.05 *vs.* CDDP group.

Data are expressed as the mean ± standard error.

## Discussion

Chemotherapy decreases the mortality of cancer patients, but it is also associated with irreversible ovarian toxicity [18,19]. Administration of a GnRH agonist has been proposed to be a non-invasive method to protect the ovarian reserve from chemotherapy, although the efficacy of this approach is still controversial [8,13,19-25]. By suppressing the hypothalamic-pituitary-ovarian axis, the GnRH agonist preserves more primordial follicles, which are not vulnerable to chemotherapeutic agents [9]. The recommended pretreatment before chemotherapy requires approximately two weeks due to the flare-up effect of gonadotropin concentration in the first week. This flare-up effect is not acceptable in two types of patients, including those suffering from rapidly progressive cancer and who cannot wait for the initiation of chemotherapy, and patients for whom an elevated gonadotropin level may promote the progress of some hormone-susceptible cancers such as breast and ovarian cancer. GnRH antagonist, which is also reported to act as a protective agent, rapidly down regulates the gonadotropin level without flare-up effect, but there are no long-acting GnRH antagonists available [9,11,26-29]. The combination of GnRH agonist and antagonist treatment is assumed to have a rapid long-acting suppression effect, avoiding the initial gonadotropin activation. Until now, four studies investigating the combination of these drugs have reported that the hormone changes are beyond those observed using controlled ovarian hyperstimulation protocols. Mardesic et al. reported a down regulation of LH 96 h after the combination treatment, and von Woff et al. found an incomplete reduction in the hormone flare-upin the combination group [12,15]. Roth et al. and Mueller et al. detected flare-up in both groups [13,14]. In our study, an increasing tendency was observed for E2, LH and FSH on day 3, but only the level of LH increased significantly. This outcome is possibly due to either the concentration peak not being observed at the right time or the animals that are not at the same stage of estrous at the beginning of treatments. Co-treatment with GnRHant in the long term or the short term significantly prevented the hormone flare-up, and the gonadotropin level rapidly fell to a low level in 5 days. This conflicting result could be attributed to differences between human and rodents, the dosage and the administration strategy. Rats have a higher concentration of GnRH receptors in the ovary, and pituitary-ovarian [19] desensitisation can be completed quickly; thus, rats may show a different response to the treatment compared to humans.

Another finding in our study was that the combination (lng) treatment significantly preserved more primordial follicles than observed in the GnRHa or GnRHant only groups. Danforth et al. reported that in a murine model, the antagonist did not protect ovaries from chemotherapy-induced toxicity, and it even depleted primordial follicles [30]. No such effect was observed in the present study, but the antagonist preserved the follicles similarly to the agonist. This result is consistent with other studies performed using the antagonist [9,11,26-29]. The combination (lng) treatment enhanced the protective effect, which was confirmed by all of our results. The combination treatment decreased the E2 serum concentration to 96.6±5.8 pg/ml (mean±SD) on day 8, and in the GnRHa and GnRHant groups, the E2 levels were 159.6±8.5 pg/ml and 151.9±10.4 pg/ml, respectively. In addition, 80% of the rats in the combination group resumed a normal oestrous cycle within 10 days after chemotherapy, while in the GnRHa and GnRHant only groups, 20% and 40% resumed a normal cycle, respectively. These results indicated that the ovarian function was maximally suppressed and quickly resumed with the combination treatment. Although Johnson et al. report that the primordial follicle pool is renewable, this characteristic remains to be confirmed [31]. Therefore, quantitative measurement of follicles is the best way to evaluate the potential fertility. Consistently, the combination treatment maximally preserved the primordial follicles. The pregnancy rate was not examined because the animals were all sacrificed for histological analysis. Nevertheless, the effect of the combination (sht) treatment was not significantly different compared to that in the GnRHa or GnRHant groups. The enhanced protective effect could be attributed to the total dosage of the two drugs without any calibration, or it could be related to the synergistic reaction between them. Our work suggests two possible methods to rapidly and effectively protect the ovary from chemotherapy-induced damage: one is to add GnRHant in the short term; the other is to increase the dosage of GnRHa. The underlying mechanism requires further investigation.

## Conclusion

In summary, our study implies that the combination of GnRH agonist with antagonist completely prevents the gonadotropin flare-up, and this combination enhances protection of the ovaries from cisplatin-induced gonadotoxicity in rats. This study may provide evidence for the application of a combination treatment to preserve fertility in female patients requiring chemotherapy.

## Competing interests

The authors declare they have no competing interests.

## Authors’ contributions

XL conceived and drafted the manuscript. XK carried out the design and performed statistical analysis of data. QD participated in the animal treatments and helped to draft manuscript. JC revised the manuscript and helped to interpret the data. ZW supervised the team and gave final approval of the version to be published. All authors read and approved the final manuscript.

## Supplementary Material

Additional file 1: Figure S1 Total tissue damage scores (mean, standard error) were significantly different among groups (P < 0.001). **P<0.01, compared with the control group. Click here for file
