# Supplementary figures and images for: Combination of a GnRH agonist with an antagonist prevents flare-up effects and protects primordial ovarian follicles in the rat ovary from cisplatin-induced toxicity: a controlled experimental animal study
Source: Reprod Biol Endocrinol. 2013 Mar 1;11:16. doi: 10.1186/1477-7827-11-16 (PMC3598983; doi:10.1186/1477-7827-11-16)

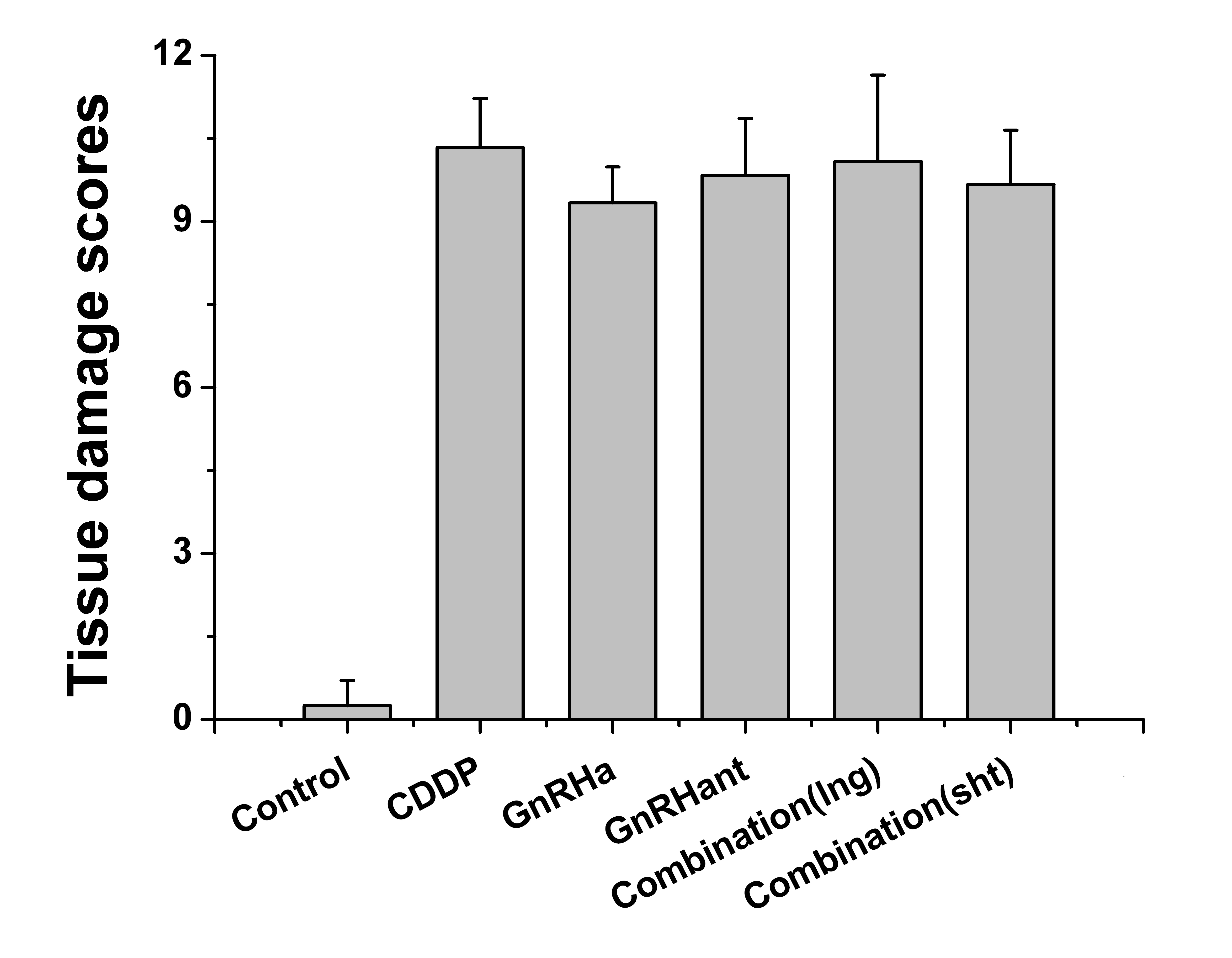

Supplement: Additional file 1: Figure S1 — Total tissue damage scores (mean, standard error) were significantly different among groups (P < 0.001). **P<0.01, compared with the control group. [file 1477-7827-11-16-S1.jpeg]
